# Epidiolex as adjunct therapy for treatment of refractory epilepsy: a comprehensive review with a focus on adverse effects

**DOI:** 10.12688/f1000research.16515.1

**Published:** 2019-02-28

**Authors:** Krithiga Sekar, Alison Pack

**Affiliations:** 1 Department of Neurology, Columbia University Irving Medical Center, New York, NY, 10032, USA

**Keywords:** cannabidiol, epilepsy

## Abstract

Medically refractory epilepsy remains an area of intense clinical and scientific interest since a significant porportion of patients continue to suffer from debilitating seizures despite available therapies. In this setting, recent studies have focused on assessing the benefits of cannabidiol (CBD)-enriched cannabis, a plant based product without psychoactive properties which has been shown to decrease seizure frequency in animal models. More recently, several randomized controlled and open label trials have studied the effects of Epidiolex, a 99% pure oral CBD extract, on patients with refractory epilepsy. This in turn has led to the FDA approval of and more recently, to the Drug Enforcement Administration’s placement of Epidiolex into schedule V of the Controlled Substances Act (CSA). In this review, we summarize the major findings of several recent large-scale studies using this product with a focus on its adverse effects.

Since up to 35% of patients with refractory epilepsy have inadequate seizure control despite currently available treatments, there has been growing interest in the use of cannabis-derived products for the treatment of medically refractory epilepsy over the past 10 years ^1, 2^. This need, combined with recent media attention focused on the benefits of non-purified cannabidiol (CBD)-enriched cannabis in the treatment of refractory epilepsy ^3^, has spurred a series of clinical trials in an attempt to more clearly understand the risks and benefits associated with this treatment.

Cannabis, derived from the plant *Cannabis sativa*, *Cannabis indica*, or *Cannabis ruderalis*, contains at least 70 known cannabinoids, including seven cannabidiolic acids and 11 tetrahydrocannabinolic acids ^4^. Tetrahydrocannabinol (THC) is a high-affinity, partial agonist of cannabinoid type 1 receptor (CB1R) and is primarily responsible for the psychotropic effects of cannabis ^5^. Though found mainly in neuron terminals of the basal ganglia, cerebellum, hippocampus, hypothalamus, and limbic system ^6^, CB1R is also present in the peripheral nervous system, thyroid, liver, uterus, and testes ^7^. Studies have shown that endogenous cannabinoids (anandamide and 2-arachidonoylglycerol) act on presynaptic CB1 receptors to downregulate both excitatory and inhibitory neurotransmitter release and thereby prevent excess neuronal activity ^7^. In contrast, exogenous THC, a partial CB1R agonist, is less selective and may inadvertently increase the release of neurotransmitters in certain brain regions ^7^, possibly giving rise to its reported pro-convulsant properties ^8^. The endocannabinoid system also contains cannabinoid type 2 receptor (CB2R), found mainly within immune cells but also seen in the spleen and the gastrointestinal tract ^7^ and on microglial cells within the central nervous system for which THC has lower affinity. Whereas studies have shown endogenous cannabinoids to enhance immune response via activation of CB2R, exogenous cannabinoids seem to have the reverse effect ^6^.

Unlike THC, CBD is non-hedonic with no known abuse potential, lacks detectable psychoactive properties, and has a relatively low affinity for both CB1R and CB2R. Despite this, CBD has surprisingly high potency as an antagonist at both of these sites ^5^ and in prior studies has been shown to counteract certain effects of THC, including tachycardia and altered cognition, when the two compounds were co-administered. This may be related in part to the finding that CBD is known to act as an “inverse agonist”, at least at the CB1 receptor site ^5^. In addition, CBD in animal studies has been shown to have anti-convulsant properties ^9^. However, the exact mechanism by which this occurs is as of yet, not fully understood. The anti-convulsant properties may not be entirely explained by the effect of CBD on CB1R and CB2R ^4^ and may be related in part to CBD-mediated modulation of the endocannabinoid system ^10^, specifically via inhibition of anandamide degradation, resulting in decreased excess neuronal activity ^11^. Alternatively, CBD has also been shown to act on multiple molecular targets which play a key role in neuronal excitability, including G protein–coupled receptor 55 (GPR55), transient receptor potential vanilloid receptors, intracellular calcium levels, and inhibitory glycine receptors ^12^. In this context, over the past several years, there has been increasing clinical, scientific, and media interest in CBD, which until recently had remained the traditionally less sought after phytocannabinoid.

Between 2013 and 2015, an increasing number of states within the US allowed access to medical cannabis. As a result, many families with individuals suffering from treatment-resistant epilepsy began to explore the use of CBD-enriched cannabis and reported successful reduction of seizure frequency ^13^. Various studies using purified CBD in animal models of partial and generalized epilepsy also showed an anti-convulsant effect ^14– 17^. Additionally, small randomized controlled trials (RCTs) and open-label studies of purified CBD in humans with treatment-resistant epilepsy had shown mixed results ^18– 20^. Around this time, significant media attention was directed toward a Colorado family who began to explore alternative treatment options for their daughter, Charlotte Figi, a child with an *SCN1A* gene mutation compatible with Dravet syndrome. After initiating adjunctive treatment with a strain of CBD-enriched cannabis (later named “Charlotte’s Web”), the Figi family reported a dramatic reduction in seizure frequency from over 300 bilateral tonic-clonic seizures per week to fewer than 30 in 3 months. By month 20, Charlotte was having two or three nocturnal convulsions a month and began to walk and talk again ^3^. By early 2014, Charlotte’s Web had reportedly been used in over 200 such cases, spurring many families to relocate to Colorado to seek out this new treatment ^3^. Interestingly, a retrospective study of oral cannabis extract used in the treatment of refractory epilepsy published during this time reported a discrepancy in responder rates between patients who had established epilepsy treatment in the state of Colorado versus patients who had newly moved to the state for this purpose. Specifically, the study showed that families who had relocated to Colorado were three times more likely to report more than 50% seizure reduction than families with established care in the state. The authors interpret this finding as representing an inherent reporting bias in those who had likely suffered significant costs and difficulty in moving there ^21^. Despite the varied findings described above, several over-the-counter CBD preparations had already become commercially available by 2015 and a US Food and Drug Administration (FDA) analysis showed that up to 33% of these contained no CBD at all ^22^. In this setting, a growing interest and need from patients, families, and physicians to better understand and regulate this product prompted the initiative to conduct several clinical trials to study CBD, ultimately facilitating the FDA approval of Epidiolex—a new, 99% pure, oral CBD extract (Epidiolex, GW Pharmaceuticals, London, UK)—in June 2018 and the Drug Enforcement Administration’s placement of Epidiolex into schedule V of the Controlled Substances Act (CSA) in September 2018. Going forward in this review, we focus on recent studies using Epidiolex to highlight what is currently known about the risks and benefits associated with this treatment modality.

Three recent randomized, multi-center, double-blinded, placebo-controlled trials ^23– 25^ using Epidiolex have been conducted and published in two epileptic syndromes primarily of childhood: the Dravet (one study) and Lennox–Gastaut (two studies) syndromes. Patients between the ages of 2 to 18 years for Dravet ^23^ and 2 to 55 years for Lennox–Gastaut ^24, 25^ with treatment-resistant epilepsy were recruited. A randomized, double-blind, placebo-controlled pilot trial was also initially completed in patients with Dravet syndrome to arrive at a target dose of 20 mg/kg per day for the phase 3 trials described above ^1^. For all three multi-center studies, a 2-week titration period was followed by 12 weeks of treatment at the target dose for a total of 14 weeks. Enrolled patients maintained a stable medication regimen (median of three anti-seizure drugs). Ketogenic diet and vagal nerve stimulator (when used) were kept stable for at least 4 weeks prior to study onset. Epidiolex target doses were 10 mg/kg given twice a day (20 mg/kg per day) for the Dravet study ^23^ and one Lennox–Gastaut study ^25^. The target doses for the additional study of Lennox–Gastaut were 5 and 10 mg/kg twice daily (10 and 20 mg/kg per day) in two treatment groups ^24^.

In patients with Dravet syndrome, a significant decrease in the median convulsive seizure (tonic, clonic, tonic-clonic, or atonic) frequency was seen in patients treated with Epidiolex when compared with placebo within the first month of the maintenance period ( *P* = 0.002) ^23^. The primary outcome endpoint was significant and showed a median reduction of 38.9% for CBD versus 13.3% for placebo ( *P* = 0.01) ^23^. The number of patients with a 50% decrease in convulsive seizures was also higher with treatment (43% versus 27% placebo), although significance was not reached ( *P* = 0.08), and three patients achieved seizure freedom with Epidiolex versus zero in the placebo group. For total seizures (that is, all seizure types), the adjusted reduction in seizure frequency was also significantly higher in the Epidiolex (28.6%) versus the placebo (9%) group ( *P* = 0.03) ^23^. Based on a Caregiver Global Impression of Change (CGIC) scale, 62% of patients in the treatment group also reported a significantly improved overall condition as compared with only 34% in the placebo group ( *P* = 0.02) ^23^.

Similarly, for patients with Lennox–Gastaut syndrome, the addition of Epidiolex to a stable regimen at doses of both 10 mg/kg per day and 20 mg/kg per day resulted in significant median percentage decreases in drop seizures (atonic, tonic, or tonic-clonic) of 41.9% for 20 mg/kg per day and 37.2% for 10 mg/kg per day versus 17% for the placebo group ( *P* = 0.005 and *P* = 0.002, respectively) ^24^. Drop-seizure reduction of at least 50% was again significantly higher in each treatment group (39% for 20 mg/kg per day and 36% for 10 mg/kg per day) compared with the placebo group (14%) ( *P* <0.001 for 20 mg/kg per day and *P* = 0.003 for 10 mg/kg per day). A dose response was seen with 25% of patients achieving a 75% reduction in drop-seizure frequency when treated with 20 mg/kg per day Epidiolex versus 11% when treated with 10 mg/kg per day of Epidiolex versus 3% receiving placebo. Median reduction of combined seizures types (convulsive and non-convulsive) was again higher in the treatment groups (38.4%, *P* = 0.009 for 20 mg/kg per day and 36.4%, *P* = 0.002 for 10 mg/kg per day) versus placebo (18.5%). Similarly, improvement in overall condition (using the CGIC scale) was significantly higher in the treatment versus placebo group ( *P* = 0.04 for 20 mg/kg per day and *P* = 0.002 for 10 mg/kg per day); interestingly, a higher percentage of patients reported improvement from baseline in the 10 mg/kg per day group (66%) versus the 20 mg/kg per day group (57%) ^24^.

The third completed phase 3 trial was a comparison of 20 mg/kg per day with placebo in patients with Lennox–Gastaut syndrome ^25^. The primary efficacy outcome of median reduction in drop seizures was significantly in favor of Epidiolex with 43.9% versus 21.8% for placebo ( *P* = 0.0135). The percentages achieving 50% drop-seizure reduction (44% for treatment versus 24% for placebo, *P* = 0.0043), median total seizure reduction (41.2% for treatment versus 13.7% for placebo, *P* = 0.0005), and improvement in the CGIC scale (58% for treatment versus 34% for placebo, *P* = 0.0012) were also significantly improved with 20 mg/kg per day of Epidiolex compared with placebo ^25^.

Additionally, several open-label, expanded-access trials studying the use of Epidiolex in patients with treatment-resistant epilepsy ^22, 26– 28^ of multiple etiologies have shown similar benefits. Specifically, median reductions in frequency by roughly 30% for motor seizures in the first 12 weeks ^22^, in the range of 50% for convulsive seizures, and up to 50 to 63% for total seizures ^22, 23, 28^ were seen in patients receiving adjunctive therapy with Epidiolex and this effect was sustained for up to 48 to 96 weeks after treatment initiation ^28^. Similarly, seizure severity was significantly improved by up to 50 to 60% with patients reporting shorter duration of seizures and post-ictal state for both pediatric and adult patients taking adjunctive Epidiolex ^26^.

The above results are encouraging and support prior preliminary findings suggesting that CBD products may indeed be a highly effective therapy for treatment-refractory epilepsy. However, the above studies and others have also demonstrated several important adverse effects associated with Epidiolex, which must also be considered as it becomes more widely used.

Randomized controlled studies in patients with Dravet or Lennox–Gastaut syndrome demonstrated that more patients in the Epidiolex versus the placebo group had adverse events (84–94% for Epidiolex versus 69–75% for placebo across the three studies) ^23– 25^ with a dose-dependent increase from 84% in patients taking 10 mg/kg per day to 94% in patients taking 20 mg/kg per day of Epidiolex ^24^. Adverse effects were most commonly seen within the first 14 days of dose escalation ^23– 25^, and in the Epidiolex group, 75% of cases were attributed to the trial agent in the Dravet study ^23^ and 62% in one Lennox–Gastaut study ^25^. The majority (78–97%) of all combined adverse events in patients receiving Epidiolex and placebo were considered mild to moderate in severity ^24, 25^. Adverse events occurring in more than 10% in any treatment group during a study across the three phase 3 trials included pyrexia, upper respiratory tract infection, somnolence, decreased appetite, diarrhea, vomiting, nasopharyngitis, status epilepticus, fatigue, convulsion, and lethargy ^23– 25^. Somnolence was reported more frequently in patients on a medication regimen that also included clobazam ^23, 25^.

Serious adverse effects also occurred more commonly in patients receiving Epidiolex versus placebo in the trials described above affecting 10 patients in the treatment versus three in the placebo group for Dravet syndrome ^23^ and 13 to 20 patients in the treatment versus 4 to 7 patients in the placebo group across the two Lennox–Gastaut studies ^24, 25^. More specifically, eight patients (Dravet study) and seven to 12 patients (Lennox–Gastaut studies) were withdrawn from the Epidiolex treatment group versus one person in the placebo group from each RCT ^23– 25^. Of note, six patients receiving 20 mg/kg per day Epidiolex were withdrawn from the trial because of adverse effects versus one patient receiving 10 mg/kg per day Epidiolex and one patient receiving placebo in one of the Lennox–Gastaut studies ^24^.

More specifically, with regard to serious adverse effects, status epilepticus was reported in similar numbers for patients receiving placebo and Epidiolex ^23, 25^. Increases in liver aminotransferase concentrations, however, were seen in patients taking Epidiolex (5–20 in the Lennox–Gastaut trials and 12 in the Dravet trial) ^23– 25^ versus one patient taking placebo in both the Dravet ^23^ and one Lennox–Gastaut ^25^ study. Again, a dose-dependent effect was seen with 11 patients in the 20 mg/kg per day Epidiolex group versus two patients in the 10 mg/kg per day group ^7^. In 79 to 80% of cases where elevated aminotransferase concentrations were greater than three times the upper limit of normal, patients were also taking concomitant valproate ^24, 25^. In all such cases, liver enzyme levels either resolved spontaneously after Epidiolex dose was decreased/stopped or the dose of another anti-seizure medication was decreased ^23– 25^. Additionally, because no significant elevation of bilirubin (greater than two times the upper limit of normal) was associated with this finding, drug-induced liver injury was not seen ^1, 24, 25^. Thrombocytopenia was also observed in patients taking both Epidiolex and valproate (3% mild to moderate) and was severe in 1% of cases with resolution after valproate was discontinued ^22^.

CBD is metabolized primarily by cytochrome P450 (CYP) 2C19 and CYP3A4 and can inhibit the CYP2C and CYP3A4 families of isoenzymes ^1^. The highest plasma concentration of CBD occurs within 2 to 3 hours of exposure to Epidiolex, and the major circulating metabolite of this compound is 7-COOH-CBD, and 6-OH-CBD is a relatively minor metabolite ^1^. Exposure to CBD (that is, in the form of Epidiolex) and its metabolites is linearly related to dose over a range of 5 to 20 mg/kg per day with high inter-subject variability in pharmacokinetics, likely related to concomitant medication interactions with the cytochrome P450 system, genetic variation in CYP isoenzymes, food effects, or tissue distribution ^1^. At doses ranging between 5 and 20 mg/kg per day, Epidiolex led to significant pharmacokinetic interactions with clobazam (CLB), resulting in elevations of N-desmethylclobazam, a long-acting active metabolite of this drug, by up to 60% via inhibition of cytochrome P450 2C19 ^1, 22, 24^. This interaction may contribute to its efficacy as adjunctive therapy and also to its associated side effect of sedation, which resolved with reduction of CLB dose in RCTs ^22^. The interaction between CLB and CBD (within Epidiolex) is thought to be bidirectional, and clobazam also increases levels of 7-OH-CBD, an active metabolite ^27, 28^. No obvious pharmacokinetic effect was seen for several other anti-seizure drugs used in combination with Epidiolex, including valproate, stiripentol, clonazepam, and levetiracetam ^1, 29^. Given that elevated aminotransferase concentrations were commonly seen with co-administration of Epidiolex and valproate without a significant increase in serum valproate drug levels, a pharmacodynamic rather than pharmacokinetic interaction has been proposed to exist between these two compounds, presumably resulting in transient metabolic stress on the liver and resulting transaminase (alanine transaminase/aspartate transaminase) elevations ^1, 23, 24^. However, the exact mechanism by which Epidiolex causes elevation in liver enzymes remains under further study. Additional studies have also shown increases in serum levels of eslicarbazepine, rufinamide, and zonisamide with adjunctive Epidiolex therapy ^29^. With regard to topiramate, studies show opposing results of no interaction versus increased serum topiramate concentrations with the addition of Epidiolex ^1, 29^. Concomitant administration of Epidiolex with warfarin showed a non-linear increase in international normalized ratio, requiring warfarin dose to be reduced by 30%, likely because of pharmacokinetic interactions involving CYP2C9 and CYP3A4, although this was reported in only one case report to date ^30^. The major RCTs using Epidiolex excluded patients who had recently started treatment with felbamate or vigabatrin (within 12 months) and thus the effect of Epidiolex in this setting requires additional data ^23– 25^.

Despite the above interactions with anti-seizure drugs, a recent open-label study showed a statistically and clinically significant decrease in overall side effects reported by patients when Epidiolex was added to their anti-seizure drug regimen. In fact, this decrease remained stable even after Epidiolex dose was increased and doses of other anti-seizure drugs were decreased ^26^. Although the authors concede that this may be related to placebo effect, a true effect is more likely since it was sustained for 48 weeks.

A common side effect reported with adjunctive treatment of Epidiolex was upper respiratory tract infections ^1, 23– 25^. Though speculative, the mechanism of this finding may be related in part to inhibition of CB2 receptors, found primarily on immune cells. CB2R activation is thought to alter cytokine release. In the central nervous system, CB2 receptors, which are found on microglia, are also thought to be involved in neurodegenerative and neuroinflammatory processes. By antagonizing CB1R/CB2R, CBD (the main compound in Epidiolex) is thought to inhibit immune cell migration and induce anti-inflammatory effects systemically and within the central nervous system. However, whether the long-term effects of this medication are favorable (anti-inflammatory) or potentially adverse in nature (increased infection/teratogenic risk) remains unknown.

Lastly, cannabinoid receptors are also found throughout the cortex, in the thyroid gland, and in human reproductive organs, including the testes and uterus ^7^. Little is known about the long-term effects of Epidiolex on cognition/memory, hormone regulation, fertility, and pregnancy. The safety profile of Epidiolex exposure *in utero* or through breast milk is similarly as of yet unknown and requires continued study as this drug becomes more widely available.

The recent FDA approval of Epidiolex combined with the placement of this compound in schedule V of the CSA (the least restrictive schedule of the CSA) has created a much-needed opportunity for the continued study of high-concentration, regulated CBD as a potential therapy for refractory epilepsy. Although recent RCTs and open-label extended-access programs have already demonstrated significant improvement in seizure frequency and severity with a relatively well-tolerated side effect profile for this compound, continued monitoring of Epidiolex is needed to further asses the long-term safety and efficacy, particularly with regard to immune, cognitive, hormonal, and reproductive function. Furthermore, there have been no large-scale RCTs demonstrating significant seizure reduction with Epidiolex in patients with focal onset seizures. Nonetheless, to date, Epidiolex has proven to be an attractive treatment option for an otherwise devastating group of epileptic syndromes. Future studies expanding our knowledge of this compound will be helpful in better understanding its role in the future of epilepsy treatment.
